# Identifying risk factors for prevalent anal human papillomavirus type 16 infection in women living with HIV

**DOI:** 10.1371/journal.pone.0268521

**Published:** 2022-05-19

**Authors:** Elaina Kaufman, Tyler Williamson, Marie-Hélène Mayrand, Ann N. Burchell, Marina Klein, Louise Charest, Sophie Rodrigues-Coutlée, François Coutlée, Alexandra de Pokomandy, on behalf of the EVVA study group

**Affiliations:** 1 Department of Family Practice, St. Paul’s Hospital Site, Faculty of Medicine, University of British Columbia, Vancouver, BC, Canada; 2 Department of Family Medicine, Faculty of Medicine, McGill University, Montreal, QC, Canada; 3 Department of Community Health Sciences, Cumming School of Medicine, University of Calgary, Calgary, AB, Canada; 4 Département d’Obstétrique-Gynécologie et Médecine Sociale et Préventive, Centre Hospitalier de l’Université de Montréal (CHUM) and Université de Montréal, Montreal, QC, Canada; 5 Department of Family and Community Medicine and MAP Centre for Urban Health Solutions, St. Michael’s Hospital, Unity Health Toronto and Department of Family and Community Medicine, Faculty of Medicine, University of Toronto, ON, Canada; 6 Chronic Viral Illness Service, McGill University Health Centre (MUHC), Montreal, QC, Canada; 7 Clinique Médicale L’Actuel, Montreal, QC, Canada; 8 Laboratoire de Virologie Moléculaire, Centre de Recherche du Centre Hospitalier de l’Université de Montréal (CHUM), CHUM et Département de Microbiologie, Infectiologie et Immunologie, Université de Montréal, Montreal, QC, Canada; Istituto Nazionale Tumori IRCCS Fondazione Pascale, ITALY

## Abstract

**Background:**

Women living with HIV (WLHIV) have a high risk of anal cancer. Identifying risk factors for anal HPV 16 infection, the most significant risk factor for anal cancer, is essential for prevention and screening strategies.

**Methods:**

In the EVVA Cohort study, 151 WLHIV had cervical and anal HPV testing with genotyping every 6 months for 2 years, while demographic and clinical data were collected via questionnaires and chart reviews. Here, we present results of baseline data analyzed using multivariable logistic regression.

**Results:**

Among 150 women with adequate HPV test results at baseline, HPV 16 DNA was detected anally in 23 (15.3%; 95%CI:10.4–22.1) and cervically in 5 (3.3%; 95%CI:1.4–7.8). In multivariable analysis, current smoking (OR = 6.0; 95%CI: 1.5–23.9), nadir CD4 count ≤ 200 cells/μL (OR = 8.4; 95%CI: 2.0–34.3), prevalent cervical HPV 16 (OR = 14.7; 95%CI: 1.0–222.5) and anogenital herpes in previous 6 months (OR = 9.8, 95%CI: 1.7–56.8) were associated with prevalent anal HPV 16.

**Conclusions:**

Knowledge of risk factors can help identify WLHIV at greatest risk of anal HPV 16 infection and, potentially, developing subsequent anal cancer. Identification of the subgroup of these women in whom HPV 16 persists could be an early step in the algorithm of anal cancer screening.

## Introduction

Among women living with HIV (WLHIV), the incidence of anal cancer is estimated at 22 cases per 100,000 person-years, in contrast to 1–2 cases per 100,000 person-years in the general population [1]. Most anal cancer research to date has focused on HIV-positive MSM due to the even higher incidence in this group [1]. Yet, there is also a need for research investigating anal cancer and related conditions in WLHIV.

Like cervical cancer, anal cancer occurs when infections with high-risk types of human papillomavirus (HR-HPV) persist, causing squamous intraepithelial lesions (SILs) [2, 3]. Akin to cervical high-grade SILs (HSILs), the precursors to invasive cervical cancer, individual anal HSILs can progress to invasive cancer [4, 5]. Although up to 22% of lesions may spontaneously regress per year [6], anal HSILs are considered the immediate precursors to invasive squamous cell carcinoma of the anus [2, 7].

Compared to other HR-HPV types, the cancer risk associated with HPV type 16 is approximately 10 times higher [3]. This high level of oncogenicity is most evident among invasive anal cancers. In one study, conducted in Québec, Canada, 92% of anal cancers were HPV-positive and 90% of these were HPV 16 positive [8]. One meta-analysis found that HPV 16 was present in over 85% of HPV-positive anal cancers [9]. Although most acquired HPV infections clear spontaneously, WLHIV are at increased risk of persistent HPV infections that can lead to oncogenic cellular changes, progression to HSIL, and, eventually, cancer [10–12]. We also know that the immune response to HPV infection is type-specific [3]. Prophylactic HPV vaccination may reduce the incidence of anal HR-HPV against the types included in the vaccine (6, 11, 16, 18, 31, 33, 45, 52, 58), but WLHIV are at increased risk of vaccine failure [13, 14]. Recent findings from a multicentre cohort study indicate that girls living with HIV mount a lower immune response to HPV vaccination than their HIV-negative peers [15]. Additionally, the immune response appears to be less robust among adult WLHIV with an unsuppressed HIV viral load (≥ 40 copies/mL), compared to fully suppressed HIV viral load (< 40 copies/mL) [16]. Risk factors for HPV are expected to differ between men and women living with HIV, due to sociocultural and anatomical differences. In addition to encouraging vaccination, we must better understand risk factors for anal HR-HPV infections, particularly HPV 16, in order to develop and implement secondary prevention strategies for anal cancer in women at highest risk [7]. To increase our understanding of the risk factors for prevalent anal HPV 16 infection, we analyzed baseline data from a cohort of WLHIV.

## Material and methods

### Study design and population

The EVVA study (“Evaluation of HIV, HPV, and AIN in Women”) is a prospective observational cohort study, which followed 151 WLHIV attending study visits every 6 months for 2 years [17, 18]. Between January 2012 and July 2015, participants were recruited during routine HIV care at four HIV clinics in Montreal, Canada (McGill University Health Centre (MUHC), Clinique Médicale l’Actuel, Centre Hospitalier de l’Université de Montréal, and Clinique OPUS). Participants met the necessary criteria for inclusion if they were aged 18 or older with confirmed positive serology for HIV, had a cervix, were not pregnant at recruitment, and had never been diagnosed with anal cancer. To be eligible, participants were required to possess sufficient knowledge of English or French to provide informed consent and understand the questionnaire. The study was approved by the research ethics boards of the McGill University Health Centre and the Centre Hospitalier de l’Université de Montréal. All participants provided informed written consent. For this analysis, which focused on prevalent anal HPV 16, we used baseline data only.

### HPV testing

Specimens for anal HPV testing were collected every 6 months by a registered nurse who used a saline-moistened Dacron swab inserted 3–5 cm into the anal canal and rotated upon removal to collect epithelial cells. The anal epithelial cell samples from swabs were agitated in 1.5 mL of PreservCyt (Hologic), a methanol-based transport medium, and stored at 4°C. Cell suspensions were centrifuged at 13,000 xg for 15 minutes at 22°C. Pellets were suspended in 300 μL of 20 mml/L Tris buffer at a pH of 8.3. The Linear Array® HPV genotyping test by Roche Molecular Diagnostics was used to detect 36 genital HPV types and the presence of β-globin [19, 20]. Samples identified as negative for β-globin and HPV were considered inadequate. HPV types considered to be HR-HPV were as follows: HPV 16, 18, 31, 33, 35, 39, 45, 51, 52, 56, 58, 59, and 68 [21].

### Questionnaires and chart reviews

Data regarding putative risk factors for anal HPV 16 infection were collected from participant questionnaires and chart review forms. Questionnaires were administered in clinic by the study coordinator, or self-administered if preferred, and included questions about sexual practices, socio-demographic characteristics, and medical history pertaining to HIV, hepatitis, HPV-related disease, HPV vaccination, anal health, sexually transmitted infections, injection drug use, and cigarette smoking. For sexually transmitted infections, participants were first asked, “Has a doctor ever told you that you had one of the following conditions? Select all that apply” The response choices included “chlamydia”, “gonorrhea”, “herpes ulcers or genital sore”, and other conditions not considered for this analysis. They were then asked, “In the last 6 months only, has a doctor told you that you had one of the following conditions? Select all that apply.”, and the same response options were provided. Chart review forms were completed based on electronic and paper patient charts by the study coordinator, documenting clinical factors including HPV vaccination, nadir CD4 count, current CD4 count, and current HIV viral load.

### Statistical analysis

Descriptive and inferential statistics were used to describe demographic and clinical characteristics. Select clinical markers, including CD4 count and HIV viral load, were dichotomized or categorized according to clinically relevant cutoffs. We focused our analyses on HPV 16 due to the higher prevalence and oncogenic potential of this HPV type for anal cancer compared to other types. Age was analyzed as a continuous linear variable, as there was no evidence of nonlinearity of its association with prevalent HPV 16. Univariable (binary) logistic regression was used to estimate odds ratios (ORs) and 95% confidence intervals (CIs) for putative correlates of prevalent anal HPV 16 infection at baseline. Risk factors to be included in the multivariable logistic regression model were selected a priori based on their potential to increase the risk of prevalent anal HPV infection. Of note, place of birth, substance use and hepatitis C infection are presented in Table 1 to better describe the study sample but were not included in the regression analyses as they are not considered direct potential risk factors for anal HPV 16. Potential correlations between variables to be used in the multivariable model were assessed using the Spearman correlation coefficient (Spearman’s ρ) and corresponding p values to verify the independence of all variables. All statistical tests were two-sided and considered significant at p<0.05. Analyses were performed using the statistical analysis software Stata/IC 14.1 for Mac (64-bit Intel).

**Table 1:** Baseline characteristics of 150 women living with HIV, by anal HPV 16 status.

Participant characteristic	n (column %)		P-value for difference
	Total (n = 150)		
	Anal HPV 16 Positive at baseline (n = 23)	Anal HPV 16 Negative at baseline (n = 127)	
**Age group** (Years)			
19–39	6 (26.1)	33 (26.0)	1.00
40–49	10 (43.5)	55 (43.3)	
50–69	7 (30.4)	39 (30.7)	
**Place of birth**			
Canada	11 (47.8)	24 (18.9)	0.02
Africa	8 (34.8)	58 (45.7)	
Caribbean	3 (13.0)	40 (31.5)	
Other	1 (4.4)	5 (3.9)	
**Education completed**			
High school or less	19 (82.6)	78 (61.4)	0.05
College or university	4 (17.4)	49 (38.6)	
**Smoking**			
Never	10 (43.5)	92 (72.4)	<0.01
Past	4 (17.4)	18 (14.2)	
Current	9 (39.1)	17 (13.4)	
**Injection drug use**			
Never	15 (65.2)	120 (94.5)	<0.01
Past	8 (34.8)	5 (3.9)	
Current	0 (0)	2 (1.6)	
**Lifetime number of vaginal sex partners**			
5 or less	11 (47.8)	83 (65.4)	0.11
More than 5	12 (52.2)	44 (34.7)	
**Ever having anal sex**			
No	16 (69.6)	94 (74.0)	0.07
Yes	7 (30.4)	33 (26.0)	
**Lifetime number of anal sex partners**			
0 or 1	16 (69.6)	117 (92.1)	<0.01
2 or more	7 (30.4)	10 (7.9)	
**Past sexual abuse/assault**			
No	8 (34.8)	70 (55.1)	0.20
Yes	15 (65.2)	53 (41.7)	
Don’t know /Prefer not to answer	0 (0)	4 (3.2)	
**Gonorrhoea (self-report)**			
Lifetime	3 (13.0)	9 (7.1)	0.33
Diagnosis in past 6 mths	0 (0)	0 (0)	n/a
**Chlamydia ever (self-report)**			
Lifetime	3 (13.0)	9 (7.1)	0.33
Diagnosis in past 6 mths	0 (0)	0 (0)	n/a
**Anogenital herpes ever (self-report)**			
Lifetime	9 (39.1)	14 (11.0)	<0.01
Diagnosis in past 6 mths	5 (21.7)	6 (4.7)	0.01
**HPV Vaccination (chart review)**			
No	23 (100.0)	121 (95.3)	0.29
Yes (at least one dose)	0 (0)	6 (4.7)	
**Hepatitis C infection, current or previous** (anti-HCV positive, from chart review)	11 (47.9)	11 (8.7)	<0.01
**CD4 cell count at baseline** (chart review)			
>200 (cells/μL of blood)	19 (82.6)	122 (96.1)	0.01
≤200 (cells/μL of blood)	4 (17.4)	5 (3.9)	
**Nadir CD4 cell count** (chart review)			
>200 (cells/μL of blood)	5 (21.7)	77 (60.6),	<0.01
≤200 (cells/μL of blood)	18 (78.3)	50 (39.4)	
**HIV viral load** (RNA copies/mL of plasma)			
Undetectable (<40)	15 (65.2)	101 (79.5)	0.07
**Prevalent Cervical HPV**			
Any HPV	20 (87.0)	55 (43.3)	<0.01
High-risk HPV	14 (60.9)	29 (22.8)	<0.01
HPV 16	4 (17.4)	1 (0.8)	<0.01

Note. HPV, human papillomavirus; High-risk HPV types include 16, 18, 31, 33, 35, 39, 45, 51, 52, 56, 58, 59, 68 [22].

## Results

Of 151 participants, the analysis included 150 women for whom adequate anal HPV testing samples had been obtained. The mean age was 45.2 years. Approximately one quarter (23%) were born in Canada, 44% in an African country, and 28% in the Caribbean. Only 2 women (1%) born in Canada self-identified as Indigenous. The vast majority (96%) responded that their preference for sexual partners was “men only”. Most women (78%) had an undetectable HIV viral load (<40 copies/mL). HPV 16 DNA was detected anally in 23 (15.3%; 95%CI:10.4–22.1) and cervically in 5 (3.3%; 95%CI:1.4–7.8) women. Among the 40 women who reported a history of anal sex, the number of lifetime anal sex partners ranged from 1 to 20. None of the 23 women with only 1 lifetime anal sex partner had prevalent anal HPV 16 (0%; 95% CI: 0–15%), compared to 7 of the 17 women who had 2 or more anal sex partners (41%; 95% CI: 18–67%). Selected baseline characteristics of the 150 participants are presented in Table 1, by anal HPV 16 status.

Findings from logistic regression analyses are presented in Table 2. In the adjusted multivariable model, current smoking (OR = 6.0; 95% CI: 1.5–23.9), nadir CD4 count ≤ 200 cells/μL (OR = 8.4; 95% CI: 2.0–34.3), prevalent cervical HPV 16 infection (OR = 14.7; 95% CI: 1.0–222.5), and anogenital herpes in previous 6 months (OR = 9.8, 95%CI: 1.7–56.8) were significantly associated with the odds of prevalent anal HPV 16 infection. As shown in Table 1, 39.1% of women with prevalent anal HPV-16 currently smoked cigarettes, 78.3% had nadir CD4 count ≤200 cells/μL, 17.4% had concurrent prevalent cervical HPV 16, and 21.7% reported anogenital herpes in the past 6 months. When looking in combination at these four identified risk factors, 8.7% (2/23) of women with prevalent anal HPV 16 had none of these risk factors; 30.4% (7/23) had only one risk factor (low nadir CD4 count for 6, and current smoking for one); and 56.5% (13/23) had two risk factors (low nadir CD4 and current smoking for 7; low nadir CD4 and prevalent cervical HPV 16 for 2; low nadir CD4 and recent anogenital herpes for 2; current smoking and recent anogenital herpes for 1; prevalent cervical HPV 16 and recent anogenital herpes for 1). Conversely, only 19 women in the entire cohort had two concomitant risk factors at baseline and, among them, the anal HPV 16 prevalence was 68.4% (13/19). Only two women in the entire cohort had three concurrent risk factors, and one of these two had prevalent anal HPV 16 (with low nadir CD4, prevalent cervical HPV 16 and recent anogenital herpes). No woman in the cohort had all four factors at baseline. Of note, 59 of the 68 women with low nadir CD4 had a baseline CD4 above 200, and the anal HPV prevalence in this group was 23.7% (14/59).

**Table 2:** Factors associated with prevalent anal HPV 16 infection.

Independent variables	Prevalent Anal HPV 16 in category	Unadjusted analysis		Adjusted model	
	n (%)	OR	95%CI	OR	95%CI
**Age at baseline in years**		1.0	1.0–1.1	1.0	0.9–1.1
19–39	6 (15.4%)				
40–49	10 (15.4%)				
50–69	7 (15.2%)				
**Smoking**					
Never	10 (9.8%)	Ref.		Ref.	
Past	4 (18.2%)	2.0	0.6–7.2	1.9	0.4–10.4
Current	9 (34.6%)	4.9	1.7–13.8	6.0	1.5–23.9
**CD4 cell count at baseline** (chart review)					
>200 (cells/μL of blood)	19 (13.5%)	Ref.		Ref.	
≤200 (cells/μL of blood)	4 (44.4%)	5.1	1.3–20.8	0.9	0.1–6.4
**Nadir CD4 cell count** (chart review)					
>200 (cells/μL of blood)	5 (6.1%)	Ref.		Ref.	
≤200 (cells/μL of blood)	18 (26.5%)	5.5	1.9–15.9	8.4	2.0–34.3
**HIV viral load at baseline** (chart review)					
< 40 copies/mL (undetectable)	15 (12.9%)	Ref.		Ref.	
≥ 40 copies/mL (detectable)	8 (23.5%)	2.1	0.8–5.4	2.1	0.6–7.7
**Prevalent Cervical HPV 16**					
No	19 (13.2%)	Ref.		Ref.	
Yes	4 (80.0%)	26.3	2.8–248.1	14.7	1.0–222.5
**Anogenital herpes in past 6 months** (self-report)					
No or don’t know	18 (13.0%)	Ref.		Ref.	
Yes	5 (45.5%)	5.6	1.5–20.3	9.8	1.7–56.8
**Lifetime number of anal sex partners**					
0 or 1	16 (12.0%)	Ref.		Ref.	
2 or more	7 (41.2%)	5.1	1.7–15.3	3.0	0.7–12.2

Note. HPV, human papillomavirus; CI, confidence interval; OR, odds ratio; Ref., reference category. The dependent variable, anal HPV 16 infection, was coded as 0 = "No HPV type 16 DNA detected in anal canal" and 1 = "HPV type 16 DNA detected in anal canal". The following variables were included in the adjusted model: age as a numerical continuous variable, smoking status, CD4 count at baseline, nadir CD4 count, HIV viral load at baseline, prevalent cervical HPV-16, self-reported history of anogenital herpes in the past 6 months, total of 2 or more partners for recent or remote anal sex.

## Discussion

These analyses based on EVVA cohort data contribute to the scant literature on risk factors for anal HPV infection in WLHIV. As for all prevalence studies, this study examined the combination of two possible outcomes represented in prevalence; the risk of acquiring HPV 16 and the risk of this infection persisting. In our cohort of WLHIV, current smoking, nadir CD4 count ≤200 cells/μL, concurrent cervical HPV 16 infection, and anogenital herpes in the last 6 months were associated with prevalent HPV 16 infection (baseline).

The anal HPV 16 prevalence of 15.3% in our study sample is relatively similar to what is observed in women living with HIV (13.9% based on data from a recent meta-analysis) and higher than what is usually measured in HIV-negative women (6.8% based on data from the same meta-analysis) [4]. In men, anal HPV 16 prevalence is 1.8% in HIV-negative men who have sex with women, 8.7% in HIV-positive men who have sex with women, 13.7% in HIV-negative men who have sex with men, and 28.5% in HIV-positive men who have sex with men [23]. Unlike some reports of risk factors for anal HPV [24] and cervical HPV in larger cohorts of WLHIV in the United States and Brazil [25], we observed little difference in HPV 16 prevalence by age. This could be due to small sample size resulting in inadequate power to detect a difference by age, but we could also observe this finding if most of our prevalent HPV 16 cases represent persistent infections rather than recent incident infections. The significance of current smoking in the multivariable model adds to ongoing discussions of the potential link between smoking and anal HPV, for which some studies in WLHIV and MSM have found no evidence [24, 26, 27] while one study in WLHIV has observed an association [28]. Like other studies in WLHIV [24, 26, 28, 29], history of anal sex (ever vs. never) was not associated with an increased odds of anal HPV 16 in our cohort. This contrasts with findings from some studies in MSM [30] and HIV-negative women [31], as well as an association with anal HSIL previously reported among HIV-positive women [32]. When anal sex history was recategorized as having two or more lifetime anal sex partners versus zero or one, the magnitude of the adjusted odds ratio increased but did not reach statistical significance. It was not possible to analyze the effect of frequency of recent anal sex (past 6 months), because only one woman with anal HPV 16 reported any recent anal sex. In contrast to a previous publication [24], we observed an association with recent anogenital herpes. Herpes may increase the odds of anal HPV 16 infection by causing mucosal ulcers and providing a potential entry point for HPV, increasing the odds of both direct anal HPV infection and autoinoculation from other anogenital sites. As an alternative hypothesis, it is possible that herpes outbreaks trigger reactivation of latent HPV 16, or that both herpes outbreaks and persistent HPV 16 infection may represent signs of lower local skin immunity. Herpes may also be more prevalent in women with higher levels of past exposure to anogenital HPV and could hence be considered a proxy variable for past exposure to sexually transmitted skin infections. Unlike a cross-sectional study conducted in France with 311 women living with HIV [33], the lifetime number of vaginal sex partners was not associated with prevalent anal HPV 16, and place of birth was no longer associated with infection with adjustment for other factors.

The strong effect of cervical HPV 16 infection in the regression model was expected, based on shared mode of acquisition, proximity of the two anatomical sites, and potential for autoinoculation. This association was previously demonstrated by Heard et al. in France [34], and a systematic review and pooled analysis by Lin et al. [4]. The consistently higher prevalence of HPV in the anus than in the cervix across studies in women may be due to a faster clearance of HPV infection at the cervix or a greater propensity of HPV detection in the anal canal to be detected due to the greater area swabbed during anal sampling.

The identification of low nadir CD4 count as a potential risk factor for anal HPV 16 is important, given the well-documented effect of nadir CD4 count on long-term health in PLHIV. Nadir CD4 count (<100 cells/μL) has been found to be associated with cervical HPV infection in WLHIV [25] and with risk of anal HSIL in MSM living with HIV [35]. While low current CD4 count at baseline was a statistically significant risk factor for prevalent anal HPV 16 in univariable analysis, the association was no longer observed when adjusted for nadir CD4 count which had a stronger effect. This suggests that immune reconstitution does not cancel the effect of reaching a low nadir CD4 count in the past, but it is unclear whether this is due to a remaining weak local immunity, or to a lower possibility of infection clearance after months or years of persistence.

This study has some limitations. We cannot distinguish persistent from new HPV 16 infections based on baseline data, recognizing that the former situation represents a higher risk of anal cancer than the latter. Some important risk factor effects might not have reached statistical significance due to type 2 error, and bias may have been introduced into the sample due to differential participation. For example, the low prevalence of certain variables (such as injection drug use) in our sample may have been due to reticence to participate among women who have experienced marginalization, and, due to the low prevalence of these variables, we were underpowered to assess them as risk factors. It is also possible that the prevalence of HPV in our sample was overestimated, particularly if women with histories of HPV-related issues were more inclined to participate. An additional limitation concerns the cross-sectional nature of the analyses: Causality cannot be inferred directly from the correlational associations we observed, despite biological plausibility that the variables identified are true risk factors. Although our sample is representative of the population of WLHIV in Québec, its generalizability to other populations may be limited, in particular due to the low number of Indigenous women and women who have a history of injection drugs.

In summary, our cross-sectional analyses confirmed the high prevalence of anal HPV 16 in WLHIV. We identified risk factors for prevalent anal HPV 16 including current smoking, nadir CD4 count ≤200, concurrent cervical HPV 16 infection, and anogenital herpes in last 6 months. Given the scarcity of studies of risk factors for prevalent anal HPV infection in WLHIV and, to our knowledge, the absence of literature specific to anal HPV 16 in this population, our findings are notable for anal cancer prevention efforts. Knowledge of risk factors for prevalent anal HPV 16 infection can help identify women at greatest risk of infection, and at increased future risk of anal precancerous and cancerous diseases. These women are most likely to benefit from screening, through either anal HPV testing, cytology, or high-resolution anoscopy.
